# LncRNA *H19* Regulates Breast Cancer DNA Damage Response and Sensitivity to PARP Inhibitors via Binding to ILF2

**DOI:** 10.3390/ijms24119157

**Published:** 2023-05-23

**Authors:** Junsong Zhao, Junchao Xu, Mingming Wu, Wei Wang, Miaomiao Wang, Leiyan Yang, Huayong Cai, Qiao Xu, Ceshi Chen, Peter E. Lobie, Tao Zhu, Xinghua Han

**Affiliations:** 1 Department of Oncology, The First Affiliated Hospital of USTC, Division of Life Sciences and Medicine, University of Science and Technology of China, Hefei 230027, China; 2 The CAS Key Laboratory of Innate Immunity and Chronic Disease, Division of Life Sciences and Medicine, University of Science and Technology of China, Hefei 230027, China; 3 Key Laboratory of Animal Models and Human Disease Mechanisms of the Chinese Academy of Sciences and Yunnan Province, Kunming Institute of Zoology, Chinese Academy of Sciences, Kunming 650201, China; 4 Tsinghua-Berkeley Shenzhen Institute and Institute of Biopharmaceutical and Health Engineering, Tsinghua Shenzhen International Graduate School, Tsinghua University, Shenzhen 518055, China; 5 Shenzhen Bay Laboratory, Shenzhen 518132, China; 6 Hefei National Laboratory for Physical Sciences, University of Science and Technology of China, Hefei 230027, China

**Keywords:** lncRNA *H19*, ILF2, breast cancer, DNA damage repair, PARP inhibitors

## Abstract

Although DNA damage repair plays a critical role in cancer chemotherapy, the function of lncRNAs in this process remains largely unclear. In this study, in silico screening identified *H19* as an lncRNA that potentially plays a role in DNA damage response and sensitivity to PARP inhibitors. Increased expression of *H19* is correlated with disease progression and with a poor prognosis in breast cancer. In breast cancer cells, forced expression of *H19* promotes DNA damage repair and resistance to PARP inhibition, whereas *H19* depletion diminishes DNA damage repair and increases sensitivity to PARP inhibitors. *H19* exerted its functional roles via direct interaction with ILF2 in the cell nucleus. *H19* and ILF2 increased BRCA1 stability via the ubiquitin-proteasome proteolytic pathway via the *H19*- and ILF2-regulated BRCA1 ubiquitin ligases HUWE1 and UBE2T. In summary, this study has identified a novel mechanism to promote BRCA1-deficiency in breast cancer cells. Therefore, targeting the *H19*/ILF2/BRCA1 axis might modulate therapeutic approaches in breast cancer.

## 1. Introduction

The DNA damage response (DDR) is an extensive signaling network involving multiple signaling pathways, such as DNA damage repair, genomic stability, cell cycle checkpoints, transcriptional regulation, and apoptosis [1,2,3]. DDR not only maintains genomic stability, its signaling network and precise regulation provides a barrier against cancer development and progression [4]. When the DDR signaling pathway is activated in response to DNA damage, the cell experiences either cell cycle arrest or apoptosis, depending on the extent of damage, thereby maintaining genomic stability and resisting cellular oncogenesis. Certain cancers possess DNA damage repair defects [5]. These defects have been exploited therapeutically, such as in the design, development and clinical application of the various poly-ADP ribose polymerase (PARP) inhibitors (PARPis) [6,7]. Double-strand breaks (DSBs) are potentially fatal DNA lesions that must be repaired to maintain chromosome integrity. Mammalian cells exhibit two main DSB repair pathways: nonhomologous end joining (NHEJ), and homologous recombination (HR)-mediated DSB repair [8]. NHEJ is regarded as mutagenic, as it may continue without a homologous DNA template [9]. In contrast, HR-mediated DSB repair yields high-fidelity repair, and requires a homologous DNA template [10,11]. Significantly, deficiencies in HR-mediated DSB repair and NHEJ are functionally associated with human cancer development and progression [2,12].

PARP is a multifunctional protease that plays a regulatory role in recognizing and repairing DNA damage, chromatin modification, transcriptional regulation, cell division, and apoptosis [13,14]. One DNA repair route that is typically suppressed in cancer is HR, a crucial mechanism of DSB repair and fork protection. Importantly, defects in the HR system produce a vulnerability that may exploit synthetic lethality to target cancer cells specifically [15]. The effectiveness of PARP inhibitors in the treatment of HR-deficient cancer has revealed a robust therapeutic strategy for a number of advanced malignancies, including ovarian and breast cancers [16]. Nevertheless, despite the encouraging results of PARPi in cancers with HR deficiencies, the paucity of therapeutic efficacy in cancer with HR sufficiency restricts its clinical utility [17,18,19]. Therefore, escalated efforts to understand the mechanism of therapeutic resistance to PARP inhibition would be helpful to improve therapeutic outcomes.

RNA transcripts that are longer than 200 nt and lack evident protein-coding potential are known as lncRNAs. LncRNAs have been reported to play a critical role in in cancer development and/or progression, either as tumor suppressors or as oncogenes [20,21,22,23]. A number of lncRNAs have been recently documented to modulate DNA damage responses, DNA repair, and genomic stability [24,25,26,27,28,29,30,31,32]. Although DDR plays a critical role in cancer chemotherapy, research on the function of long noncoding RNAs (lncRNA) in cancer cell responses to cytotoxic agents remains rather limited. In this study, in silico cross-screening of NGS data for lncRNAs associated with DNA damage response and PARP inhibitor resistance was performed, demonstrating that long noncoding RNA *H19* regulates both DDR and sensitivity to PARPis in breast cancer.

## 2. Results

### 2.1. H19 Is Associated with DNA Damage in Breast Cancer

**Figure 1:**
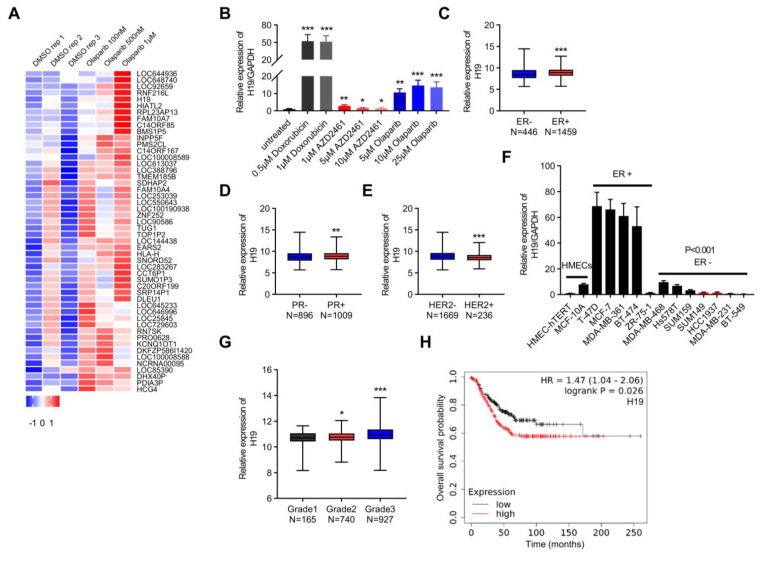
*H19* is associated with DNA damage in breast cancer. (**A**) The heatmap lists differentially expressed lncRNAs in the GEO database (GSE56400). (**B**) The *H19* levels in response to Doxorubicin and PARP inhibitor treatment were determined by qRT-PCR. Data are shown as means ± SD. * *p* < 0.05, ** *p* < 0.01, and *** *p* < 0.001 by two-tailed Student’s *t*-test. Data are representative of at least three independent experiments. (**C**) Box plot showing *H19* expression in ER positive (*N* = 1459) breast cancer samples versus ER negative (*N* = 446) samples. *** *p* < 0.001 by two-tailed Student’s *t*-test. (**D**) Box plot showing *H19* expression in PR positive (*N* = 1009) breast cancer samples versus PR negative (*N* = 896) samples. ** *p* < 0.01 by two-tailed Student’s *t*-test. (**E**) Box plot showing *H19* expression in HER2 negative (*N* = 1669) breast cancer samples versus HER2 positive (*N* = 236) samples. *** *p* < 0.001 by two-tailed Student’s *t*-test. (**F**) The *H19* levels in 14 human mammary cell lines were determined by qRT-PCR. Data are shown as means ± SD. *p* < 0.001 by two-tailed Student’s *t*-test. Data are representative of at least three independent experiments. (**G**) Box plot showing *H19* expression across different breast cancer histological grading in the TCGA breast cancer cohorts. * *p* < 0.05, and *** *p* < 0.001 by two-tailed Student’s *t*-test. (**H**) Overall survival of breast cancer patients based on *H19* expression in the KM-Plotter database. The Affymetrix ID of *H19* is 224646_x_at. The upper quartile expression value was used as the cutoff. *p* value was determined by the Log-rank test.

To identify lncRNAs associated with DDR and PARP inhibitor resistance, we analyzed the GSE56400 file in the Gene Expression Omnibus (GEO) database [33]. By analyzing the differentially expressed lncRNAs between the groups treated with DMSO or Olaparib (a small molecule inhibitor of PARP1/PARP2), a total of 704 differentially expressed lncRNAs were identified. *H19* was observed as one of the top five differentially expressed lncRNAs, suggesting possible involvement in DNA damage (Figure 1A). *H19* expression was assessed using qRT-PCR in MCF-7 (estrogen receptor positive: ER+) breast cancer cells treated with Doxorubicin (a DNA intercalating agent and potent Topoisomerase II inhibitor used to induce DNA double-strand breaks) or PARPis (Figure 1B), and increased *H19* expression was observed in the Doxorubicin or PARPi-treated group. The correlation of *H19* expression with the clinicopathologic features of breast cancer was further examined using The Cancer Genome Atlas (TCGA) dataset. Consistently, a significant high expression of *H19* in ER-positive specimens was observed compared with ER-negative breast cancer tissues (Figure 1C). Higher *H19* levels were correlated with PR positivity in breast cancer samples (Figure 1D). In comparison, *H19* levels exhibited a negative correlation with HER2 status (Figure 1E). The expression levels of *H19* in a set of 14 different *human* mammary cell lines were assessed as well. Consistently, a higher level of *H19* expression was observed in ER+ breast cancer cell lines compared with ER negative breast cancer cell lines (Figure 1F). It was observed that *H19* expression was associated with the histological grade of breast cancer (Figure 1G). Furthermore, increased *H19* expression was observed in patients with a poor prognosis and was correlated with the overall survival of breast cancer patients in the Kaplan–Meier Plotter database (Figure 1H). Thus, these findings imply that *H19* may modulate the DNA damage response of breast cancer cells.

### 2.2. H19 Regulates DNA Damage Response and Sensitivity to PARP Inhibitors

**Figure 2:**
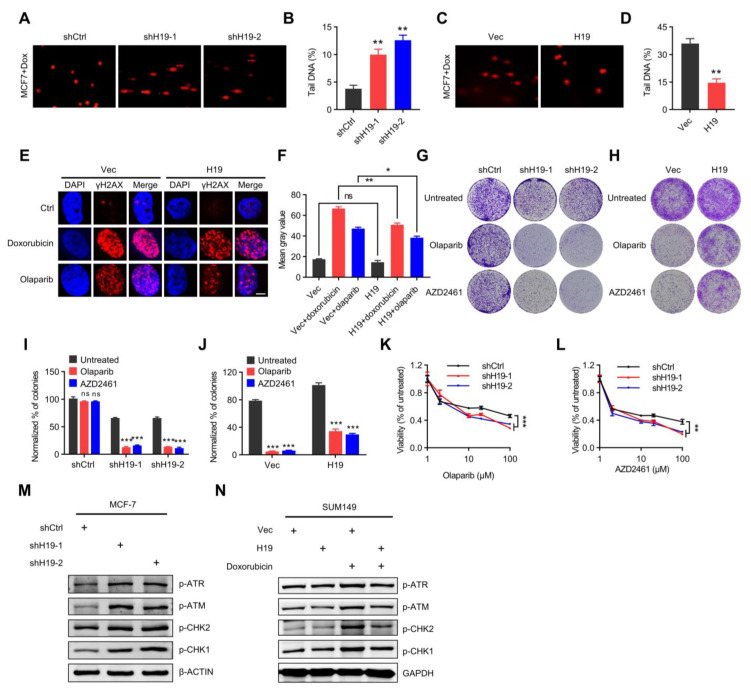
*H19* regulates DNA damage response and sensitivity to PARP inhibitors. (**A**,**B**) Doxorubicin-induced DNA damage in control and *H19*-depleted MCF-7 cells as measured by comet assay. Scale bars, 10 µm. Levels of DNA damage quantified by the tail moment. Error bars, s.d. ** *p* < 0.01 by two-tailed Student’s *t*-test. *n* = 3 independent cell cultures. (**C**,**D**) Doxorubicin-induced DNA damage in control and MCF-7 cells with forced expression of *H19* as measured by comet assay. Scale bars, 10 µm. Levels of Doxorubicin-induced DNA damage quantified by the tail moment. Error bars, s.d. ** *p* < 0.01 by two-tailed Student’s *t*-test; *n* = 3 independent cell cultures. (**E**) Doxorubicin or Olaparib-induced DNA damage in control and T47D cells with forced expression of *H19* as measured by immunofluorescence staining. Representative pictures of γ-H2AX-positive foci in control cells and cells with forced expression of *H19*. Scale bars, 5 µm. One hundred cells were analyzed for each time point. (**F**) Quantification of the mean grey value of γ-H2AX-positive foci in the control cells and T47D cells with forced expression of *H19*. Error bars, s.d. ns, not significant, * *p* < 0.05, and ** *p* < 0.01 by two-tailed Student’s *t*-test; *n* = 3 independent cell cultures. (**G**) Long-term clonogenic assay using MCF-7 cells transfected with the indicated constructs and treatments. (**H**) Long-term clonogenic assay using SUM149 cells transfected with the indicated constructs and treatments. (**I**) Quantification of the clonogenic assay in G by determining the absorbance of crystal violet at 590 nm. All groups were normalized to the absorbance of the vector control. The data represent the mean ± s.d. ns, not significant, and *** *p* < 0.001 by two-tailed Student’s *t*-test; *n* = 3 independent cell cultures. (**J**) Quantification of the clonogenic assay in H by determining the absorbance of crystal violet at 590 nm. All the groups were normalized to the absorbance of the vector control. The data represent the mean ± s.d. *** *p* < 0.001 by two-tailed Student’s *t*-test; *n* = 3 independent cell cultures. (**K**) Survival of control or *H19*-depleted MCF-7 cells in response to Olaparib treatments. Cells transfected with control or *H19*-specific shRNAs were treated with different concentrations of Olaparib and the survival after treatment was measured. Error bars, s.d. *** *p* < 0.001 by ANOVA test; *n* = 3 independent cell cultures. (**L**) Survival of control or *H19*-depleted MCF-7 cells in response to AZD2461 treatment. Cells transfected with control or *H19*-specific shRNAs were treated with different concentrations of AZD2461 and the survival after treatment was measured. Error bars, s.d. ** *p* < 0.01 by ANOVA test; *n* = 3 independent cell cultures. (**M**) Several DNA damage response-related proteins were detected in *H19*-depleted and shCtrl MCF-7 cells. Cell lysates were analyzed by immunoblotting. The data shown represent three independent experiments. (**N**) Several DNA damage response-related proteins were detected in *H19* forced expression and control SUM149 cells pre-treatment with doxorubicin. Cell lysates were analyzed by immunoblotting. The data shown represent three independent experiments.

Considering that *H19* is associated with poor prognosis and response to PARP inhibition in breast cancer, it was logically hypothesized that *H19* might be involved in DSB repair. Using comet assays, the effect of *H19* depletion on the repair of Doxorubicin-induced DNA damage in MCF-7 cells was evaluated. Twelve hours after Doxorubicin treatment, the degree of DNA damage progressively reverted to baseline in control cells and remained elevated in *H19* depleted cells, indicating that DNA repair was delayed as a result of *H19* depletion by shRNA (Figure 2A,B and Figure S1A). In contrast, forced expression of *H19* resulted in reduced Doxorubicin-induced DNA damage in MCF-7 cells compared to the control cells (Figure 2C,D and Figure S1B). Collectively, these observations suggest that *H19* promotes DSB repair activity.

When DNA damage occurs in cells, punctate signaling aggregates of phosphorylated histone H2AX (γH2A.X) and p53 binding protein 1 (53BP1) are formed, representing the recruitment of DDR components to damage sites [34]. Hence, phosphorylated histone H2AX (γ-H2AX) levels after Doxorubicin or Olaparib treatment of breast cancer cells were examined. Compared with control cells, cells with forced expression of *H19* exhibited lower levels of γ-H2AX (Figure 2E,F). Consistently, similar results were observed as to the differences in 53BP1 levels after Doxorubicin or Olaparib treatment. Compared with control cells, cells with forced expression of *H19* exhibited lower levels of 53BP1 (Figure S1C,D). These results suggest that *H19* facilitates cellular DNA damage repair and the maintenance of genomic stability. Long-term clonogenic assays revealed that *H19* depletion resulted in increased sensitivity of BRCA1 wild-type MCF-7 cells to Olaparib and AZD2461 (a small molecule inhibitor of PARP1/PARP2) (Figure 2G,I). Forced expression of *H19* resulted in reduced sensitivity of BRCA1 mutant SUM149 cells to Olaparib and AZD2461 (Figure 2H,J). Consistently, *H19*-depleted MCF-7 cells exhibited increased sensitivity to Olaparib and AZD2461 compared to the control cells (Figure 2K,L). Hence, it is apparent that *H19* reduces sensitivity to PARP inhibition in breast cancer cells.

The expression of DDR markers in *H19*-depleted MCF-7 cells was further examined. *H19* depletion increased the phosphorylated levels of ATM, ATR, CHK1, and CHK2 (Figure 2M). The expression of DDR markers in BRCA1 mutant SUM149 cells with forced expression of *H19* and treated with Doxorubicin was examined as well. It was observed that forced expression of *H19* abrogated the increased phosphorylation levels of ATM, ATR, CHK1, and CHK2 afforded by Doxorubicin treatment (Figure 2N). These results suggest that *H19* promotes the breast cancer DNA damage response by affecting DNA damage repair signaling pathways.

### 2.3. H19 Interacts with ILF2 Directly

**Figure 3:**
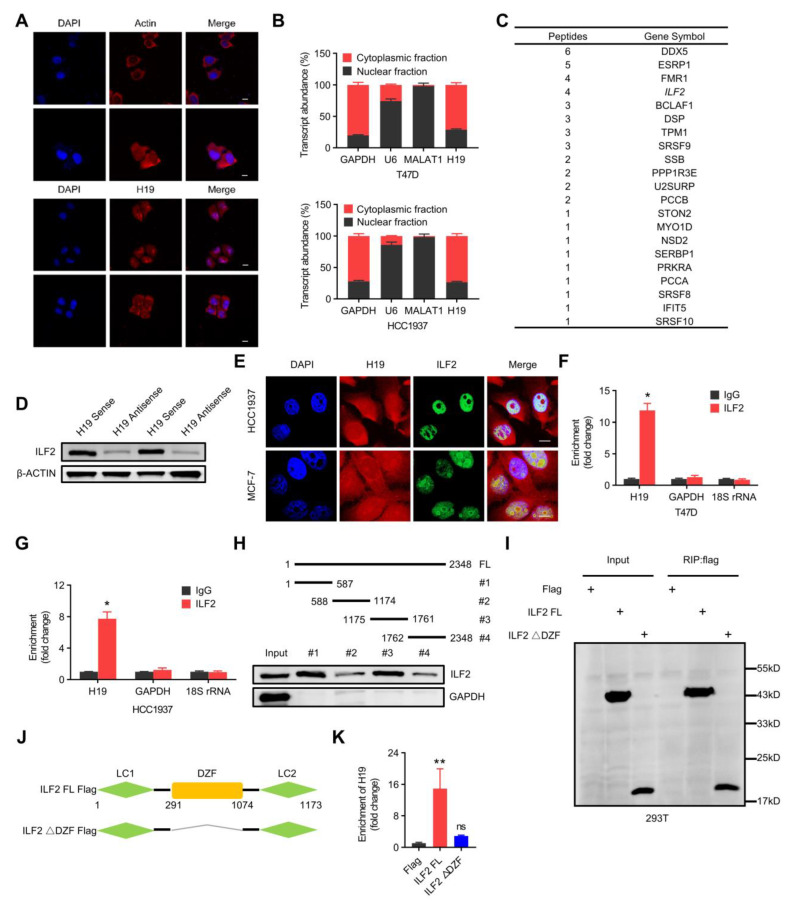
*H19* interacts with ILF2 directly. (**A**) *H19* intracellular localization was visualized in MCF-7 cells by RNA-FISH assays. Representative images are shown. DAPI, 4′,6-diamidino-2-phenylindole. Probes, *H19*. Scale bar, 10 μm. (**B**) Subcellular fractionation of T47D and HCC1937 cells followed by quantitative real-time PCR. U6 RNA served as a positive control for nuclear gene expression. Data are shown as means ± SD. Data are representative of at least three independent experiments. (**C**) Biotin-RNA pulldowns were performed with whole extracts of MCF-7 cells using full-length *H19* transcript (sense) and antisense followed by mass spectrometry. This table shows the differential proteins that were enriched. (**D**) The interaction between *H19* and ILF2 was confirmed by RNA pulldown and western blot. β-ACTIN was used as input control. (**E**) *H19* was visualized by RNA-FISH, and immunofluorescence staining of ILF2 in MCF-7 and HCC1937 cells was performed. Scale bar, 10 μm. (**F**,**G**) The interaction of *H19* with ILF2 was verified by an RNA immunoprecipitation (RIP) assay. The results are shown as means ± SD. * *p <* 0.05 by two-tailed Student’s *t*-test. (**H**) Mapping analysis of ILF2 binding domains of *H19*, showing the following: schematic diagram of *H19* full-length and truncated fragments (**top panel**); western blot of ILF2 in RNA pulldown samples by different *H19* fragments (**bottom panel**). (**I**–**K**) Deletion mapping for the domains of ILF2 that bind to *H19*. RIP analysis for *H19* enrichment in cells transiently transfected with plasmids containing the indicated FLAG-tagged full-length or truncated constructs. The results are shown as means ± SD. ns, not significant, and ** *p* < 0.01 by two-tailed Student’s *t*-test; *n* = 3 independent cell cultures.

The nuclear and cytoplasmic localization of *H19* was determined by use of RNA fluorescence with in situ hybridization (RNA-FISH) in MCF-7 cells and cellular fractionation in T47D and HCC1937 breast cancer cells (Figure 3A,B). *H19* was detected in both the cytoplasm and nucleus. For further mechanistic insight, RNA pulldown assays followed by mass spectrometry were employed to determine *H19* interacting proteins. A list of candidate proteins potentially interacting with *H19* was screened further using western blot analysis. Of those candidate proteins, only ILF2 was verified to be precipitated with biotin-labeled sense *H19* (Figure 3C,D). ILF2 is implicated in the DDR process that was exclusively detected in *H19*-associated samples [35]. High signals for ILF2 in proteins brought down with biotin-labeled sense *H19* were observed, but were not seen in proteins associated with biotin-labeled antisense *H19*, confirming ILF2 enrichment in the *H19*-related protein complex. Moreover, *H19* was colocalized with ILF2 in the nucleus of MCF-7 and HCC1937 cells (Figure 3E). These data indicate that *H19* is associated with ILF2 in the nuclei of breast cancer cells. Moreover, the possible interaction between ILF2 and *H19* was reciprocally detected by RIP using ILF2 antibody (Figure 3F,G). Relative to the IgG-bound complexes, ILF2-specific antibody-bound complexes contained considerably more *H19* RNA. In addition, no enrichment by the negative control GAPDH or 18S rRNA was observed in complexes immunoprecipitated with ILF2-specific antibodies.

A series of *H19* truncations were constructed to map its binding fragment with ILF2, and it was observed that the two fragments of *H19* (regions #1 and #3) were sufficient for binding to ILF2 (Figure 3H). This result confirmed the specific binding of *H19* and ILF2 via regions #1 and #3 on *H19*. For reciprocal confirmation, a series of ILF2 truncations were constructed to map its *H19*-interacting regions. It was revealed that the DZF domain of ILF2 was critical in the interaction between ILF2 and *H19* (Figure 3I–K). The data above demonstrates that *H19* interacts with ILF2 directly.

### 2.4. H19 Regulates DNA Damage Repair via Binding to ILF2

**Figure 4:**
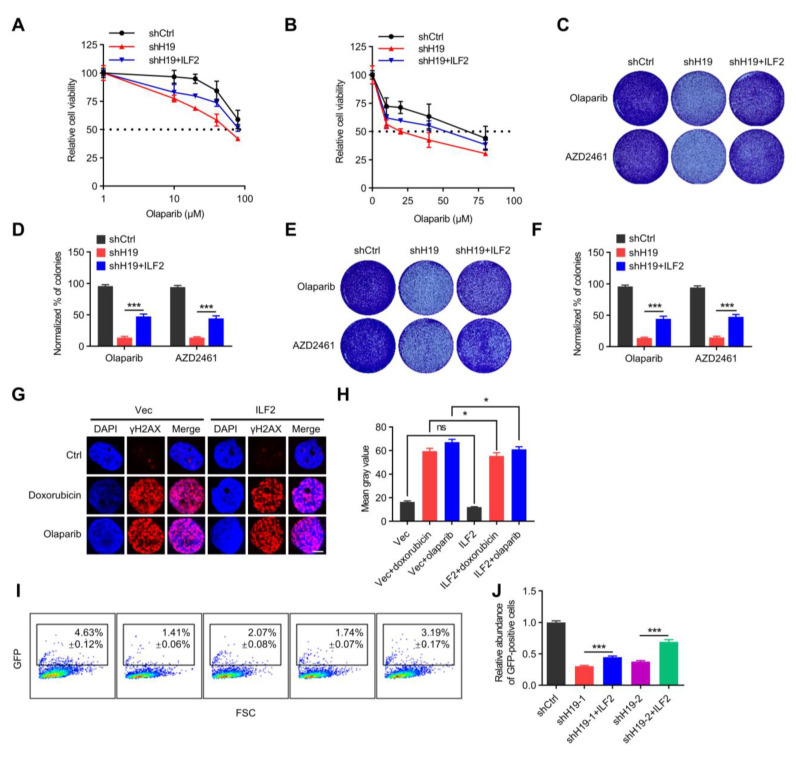
*H19* regulates DNA damage repair via binding to ILF2. (**A**) Cell viability of MCF-7 cells transfected with *H19* shRNA and/or Flag-ILF2 was determined by MTT. (**B**) Cell viability of T47D cells transfected with *H19* shRNA and/or Flag-ILF2 was determined by MTT. (**C**) Colony formation was determined in MCF-7 cells transfected with *H19* shRNA and/or Flag-ILF2. (**D**) Quantification of the clonogenic assay in C by determining the absorbance of crystal violet at 590 nm. All the groups were normalized to the absorbance of the vector control. The data represent the mean ± s.d. *** *p* < 0.001 by two-tailed Student’s *t*-test; *n* = 3 independent cell cultures. (**E**) Colony formation was determined in T47D cells transfected with *H19* shRNA and/or Flag-ILF2. (**F**) Quantification of the clonogenic assay in E by determining the absorbance of crystal violet at 590 nm. All groups were normalized to the absorbance of the vector control. The data represent the mean ± s.d. *** *p* < 0.001 by two-tailed Student’s *t*-test; *n* = 3 independent cell cultures. (**G**) Doxorubicin or Olaparib-induced DNA damage in control and T47D cells with forced expression of ILF2 as measured by immunofluorescence staining. Representative pictures of γ-H2AX-positive foci in control cells and cells with forced expression of ILF2. Scale bars, 5 µm. One hundred cells were analyzed for each time point. (**H**) Quantifying the mean grey value of γ-H2AX-positive foci in control cells and cells with forced expression of ILF2. Error bars, s.d. ns, not significant, and * *p <* 0.05 by two-tailed Student’s *t*-test; *n* = 3 independent cell cultures. (**I**,**J**) *H19*-depletion or ILF2-supplementation modulated HR DNA repair. FSC, forward scatter. Data are shown as mean ± s.d. *** *p* < 0.001 by two-tailed Student’s *t*-test; *n* = 3 independent experiments.

To further determine whether ILF2 mediated *H19*-elicited functionality, *H19* was depleted in MCF-7 and T47D cells with concomitant forced expression of ILF2. The data showed that forced expression of ILF2 partially restored *H19* depletion-reduced cell sensitivity to PARP inhibition (Figure 4A,B). Using long-term clonogenic experiments, it was observed that forced expression of ILF2 partially restored the reduced cell sensitivity to PARP inhibition afforded by *H19* depletion in BRCA1 wild-type MCF-7 and T47D cells (Figure 4C–F). Phosphorylated histone H2AX (γ-H2AX) levels after Doxorubicin or Olaparib treatment were further examined. Compared with control cells, cells with forced expression of ILF2 exhibited lower levels of γ-H2AX (Figure 4G,H). Consistently, cells with forced expression of ILF2 exhibited lower levels of 53BP1 after Doxorubicin or Olaparib treatment compared with control cells (Figure S1E,F). In addition, *H19* was depleted in BRCA1 wild-type MCF-7 cells, with concomitant forced expression of ILF2. The data showed that forced expression of ILF2 abrogated the increased cell sensitivity to PARP inhibition afforded by *H19* depletion (Figure S1G,H).

In addition, whether *H19* is involved in HR repair was assessed. To establish MCF7-DR-GFP (MCF7-HR) cells, the pDR-GFP plasmid was stably integrated into MCF7 cell lines [36]. The introduction of I-sceI expression constructs into MCF7-HR cells provides a measure of the respective HR repair pathways as measured by green fluorescent protein (GFP)-positive cells [37]. Notably, cells that were depleted of *H19* displayed a marked reduction in the number of GFP-positive cells in MCF7-HR assays. Through HR repair assays, it was observed that forced expression of ILF2 partially restored the reduced HR repair ability afforded by *H19* depletion (Figure 4I,J). Therefore, it is apparent that *H19* regulates breast cancer cell sensitivity to PARP inhibition via binding to ILF2.

### 2.5. H19 and ILF2 Affect the Expression of Genes Involved in DNA Damage Response

**Figure 5:**
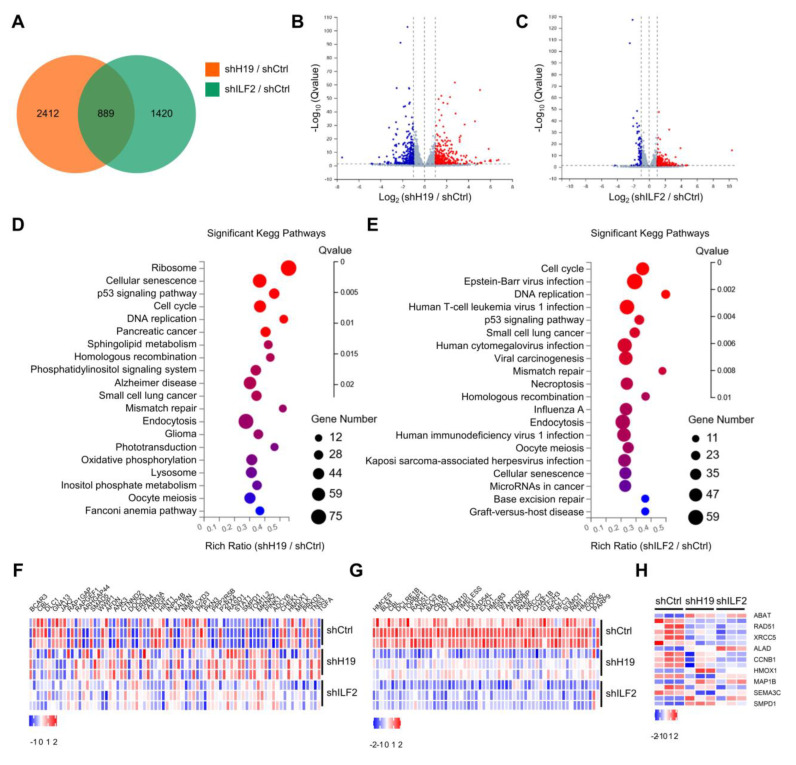
*H19* and ILF2 affect the expression of genes involved in the DNA damage response. (**A**) Venn diagram representing 889 overlapping transcripts obtained from *H19* RNA-seq (yellow) and ILF2 RNA-seq (green). (**B**) Volcano plot showing differentially expressed mRNAs in MCF-7 cells upon *H19* depletion. (**C**) Volcano plot showing differentially expressed mRNAs in MCF-7 cells upon ILF2 depletion. (**D**) Summary of gene set enrichment analysis (GSEA) of protein-coding genes ranked by differentially expressed genes (*H19* depletion versus control) correlation values using annotated KEGG (Kyoto Encyclopedia of Genes and Genomes) pathways. (**E**) Summary of gene set enrichment analysis (GSEA) of protein-coding genes ranked by differentially expressed genes (ILF2 knockdown versus control) correlation values using annotated KEGG (Kyoto Encyclopedia of Genes and Genomes) pathways. (**F**) Differential HR-related genes were validated in *H19* or ILF2-silenced MCF-7 cells. (**G**) Differential DDR-related genes were validated in *H19* or ILF2-silenced MCF-7 cells. (**H**) Differential responses to drug-related genes were validated in *H19* or ILF2-silenced MCF-7 cells.

To determine target genes of *H19* and ILF2, *H19*-silenced and ILF2-silenced MCF-7 cells were established to perform transcriptome sequencing analyses. *H19* depletion resulted in altered expression of 3301 genes, while ILF2 depletion led to differential expression of 2309 genes. Examining their intersection, *H19* and ILF2 concurrently influenced the expression of 889 genes (Figure 5A). Moreover, differential gene expression profiles revealed significantly downregulated or upregulated transcripts in *H19*-silenced or ILF2-silenced MCF-7 cells (Figure 5B,C).

Pathway enrichment analysis suggested that cell cycle, homologous recombination, and mismatch repair were among the most enriched pathways in *H19*-silenced or ILF2-silenced MCF-7 cells (Figure 5D,E). Furthermore, certain differentially expressed genes were enriched in homologous recombination signaling pathways. Notably, *H19* or ILF2 depletion significantly reduced the expression of several homologous recombination related genes, such as BRCA1, MRE11, EXO1, and RAD51, among others (Figure 5F). These data indicate that *H19* and ILF2 possess pivotal roles in homologous recombination. Gene set enrichment analysis (GSEA) suggested that *H19* and ILF2 affected gene expression of the p53 signaling pathway and UV response (Figure S2G,H). Notably, *H19* depletion dramatically downregulated the expression of several DDR-related molecules, including BRCA1, MRE11, XRCC3, and CHEK1. (Figure 5G). These findings suggest that *H19* maintains the DDR signaling pathway. In addition, GSEA analysis suggested that depletion of *H19* or ILF2 affected the expression of genes related to drug transporters and Doxorubicin resistance (Figure S2E,F). It was found that *H19* silencing dramatically downregulated expression of several drug responses genes, including ABAT, DVL3, and STAT3 (Figure 5H).

### 2.6. H19 and ILF2 Increase the Expression and Stability of BRCA1

**Figure 6:**
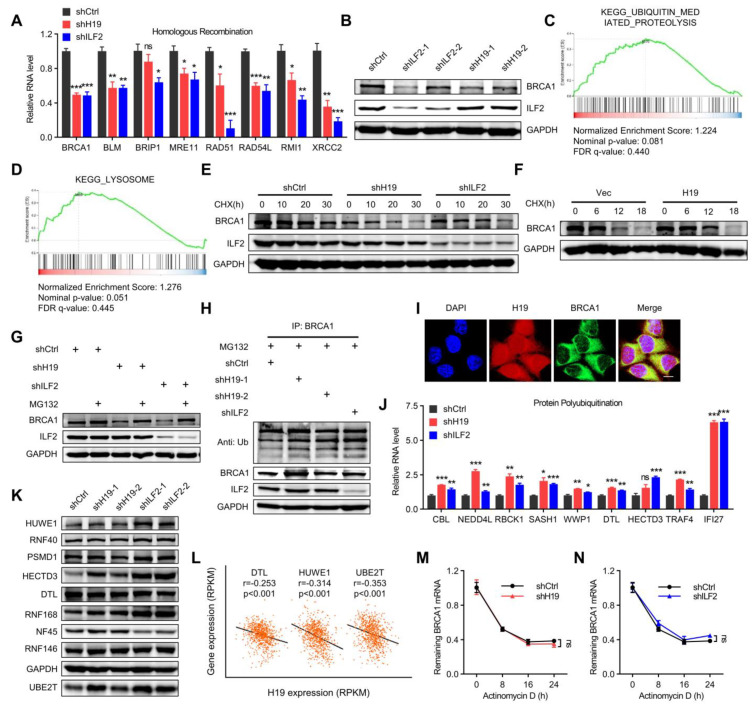
*H19* and ILF2 increase the expression and stability of BRCA1. (**A**) Differential homologous recombination-related genes were validated in *H19*- or ILF2-silenced MCF-7 cells. Data are shown as means ± SD. ns, not significant, * *p* < 0.05, ** *p* < 0.01, and *** *p* < 0.001 by two-tailed Student’s *t*-test. Data are representative of at least three independent experiments. (**B**) BRCA1 was detected in *H19*-depleted, ILF2-depleted, and shCtrl MCF-7 cells. (**C**) GSEA plots of ubiquitin-mediated proteolysis-related signatures in *H19*-depleted or ILF2-depleted MCF-7 cells versus control cells. FDR, false discovery rate; NES, normalized enrichment score. (**D**) GSEA plots of lysosome degradation pathway-related signatures in *H19*-depleted or ILF2-depleted MCF-7 cells versus control cells. FDR, false discovery rate; NES, normalized enrichment score. (**E**) MCF-7 cells with stable depletion of *H19* or ILF2 and control cells were treated with cycloheximide (CHX; 20 µg/mL) or vehicle for the indicated periods. BRCA1 levels were analyzed by immunoblotting. (**F**) SUM149 cells with stable forced expression of *H19* and control cells were both treated with cycloheximide (CHX; 20 µg/mL) or vehicle for the indicated periods. BRCA1 levels were analyzed by immunoblotting. (**G**) MCF-7 cells with stable depletion of *H19* or ILF2 and control cells were treated with MG132 (5 µM) or vehicle for 24 h. Cell lysates were analyzed by immunoblotting. (**H**) MCF-7 cells with stable forced expression of *H19* or depletion of *H19* were treated with MG132 (5 µM) for 24 h. Cell lysates were immunoprecipitated (IP) with either control IgG or antibody against BRCA1 and analyzed by immunoblotting with ubiquitin (Ub)-specific antibody. Bottom, input from cell lysates. (**I**) *H19* was visualized by RNA-FISH and immunofluorescence staining of BRCA1 in MCF-7 cells was performed. Scale bar, 10 μm. (**J**) Differential protein polyubiquitination-related genes were validated in *H19*- or ILF2-silenced MCF-7 cells. Data are shown as means ± SD. ns, not significant, * *p* < 0.05, ** *p* < 0.01, and *** *p* < 0.001 by two-tailed Student’s *t*-test. Data are representative of at least three independent experiments. (**K**) Several E3 ubiquitin ligases were detected in *H19*-depleted, ILF2-depleted, and shCtrl MCF-7 cells. Cell lysates were analyzed by immunoblotting. (**L**) Pearson correlation between *H19* expression and expression of DTL, HUWE1, and UBE2T in the breast cancer samples (817 samples in which *H19* expression was detectable were used in correlation analysis). (**M**) MCF-7 cells expressing control shRNA or *H19* shRNA were treated with actinomycin D (1 µg/mL) for the indicated periods. Total RNA was purified and then analyzed by real-time RT-PCR to examine the mRNA half-life of BRCA1. Data shown are the mean ± SD. ns, not significant by ANOVA test; *n* = 3 independent experiments. (**N**) MCF-7 cells expressing control shRNA or ILF2 shRNA were treated with actinomycin D (1 µg/mL) for the indicated periods. Total RNA was purified and then analyzed by real-time RT-PCR to examine the half-life of BRCA1 mRNA. Data shown are the mean ± SD. ns, not significant by ANOVA test; *n* = 3 independent experiments.

BRCA1 was subsequently verified as a downstream gene of *H19* or ILF2 (Figure 6A). Consistently, BRCA1 protein levels decreased in *H19*-depleted or ILF2-depleted cells (Figure 6B). GSEA analysis suggested that *H19* or ILF2 affected the gene expression of the ubiquitin-mediated proteolysis and lysosome degradation pathways (Figure 6C,D). BRCA1 stability has been reported to be regulated by the ubiquitin-proteasome pathway [38,39]. Therefore, whether *H19* and/or ILF2 affected BRCA1 protein stability was determined. A significantly shorter half-life of BRCA1 was observed in *H19* or ILF2-depleted MCF-7 cells compared to the control cells (Figure 6E). In addition, a significantly longer half-life of BRCA1 was observed in SUM149 cells with forced expression of *H19* compared to the control cells (Figure 6F). Furthermore, *H19* or ILF2-depleted MCF-7 cells were treated with the proteasome inhibitor MG132. The data indicated that MG132 treatment restored *H19* and that ILF2 depletion decreased BRCA1 expression (Figure 6G). Hence, the ubiquitination of BRCA1 in *H19*- or ILF2-depleted MCF-7 cells was determined. BRCA1 ubiquitination was increased in *H19*- or ILF2-depleted MCF-7 cells (Figure 6H). These data suggest that *H19* and ILF2 regulate BRCA1 stability via the ubiquitin-proteasome proteolytic pathway. *H19* was colocalized with BRCA1 in the MCF-7 cell nucleus (Figure 6I). Furthermore, differential protein polyubiquitination-related genes were verified by qRT-PCR analysis in *H19*- or ILF2-silenced MCF-7 cells (Figure 6J).

The mechanism by which *H19* and ILF2 regulate BRCA1 stability was investigated. Thus, it was determined whether *H19* and ILF2 could affect the expression levels of ubiquitin ligases. Notably, *H19* or ILF2 depletion resulted in increased expression of several ubiquitin ligases, including HUWE1 and UBE2T (Figure 6K). The correlation between *H19* and several E3 ubiquitin ligases in the TCGA database were examined. *H19* expression was negatively correlated with DTL, HUWE1, and UBE2T (Figure 6L). HUWE1 and UBE2T have been reported to interact with BRCA1, and function as E3 ubiquitin ligases [38,39]. These results suggest that depletion of *H19* or ILF2 increases BRCA1 ubiquitination, possibly by upregulation of HUWE1 and UBE2T. To evaluate the possibility that *H19* and ILF2 may affect the stability of BRCA1 mRNA, MCF-7 cells were treated with actinomycin D. The half-life of BRCA1 mRNA was unaffected by *H19* or ILF2 depletion (Figure 6M,N). These results demonstrate that *H19* and ILF2 stabilize BRCA1 without affecting the half-life of BRCA1 mRNA.

### 2.7. H19 and ILF2 Confer Resistance to PARP Inhibitors In Vivo

**Figure 7:**
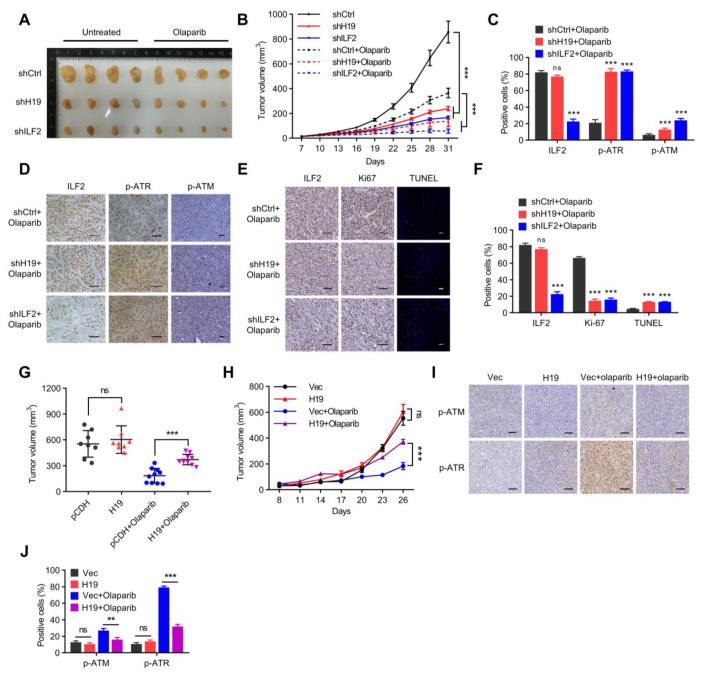
*H19* and ILF2 confer resistance to PARP inhibitors in vivo. (**A**) Final sizes of individual MCF-7-derived *H19*-silenced, ILF2-silenced, and shCtrl xenografts treated with a regimen of 50 mg/kg Olaparib daily for 28 days or left untreated. (**B**) Relative xenograft growth of individual MCF-7-derived *H19*-silenced, ILF2-silenced, or shCtrl xenografts treated with a regimen of 50 mg/kg Olaparib daily for 28 days or left untreated. Data shown are the mean ± SD. *** *p* < 0.001 by ANOVA test; *n* = 4 mice for each group. (**C**) Quantification of ILF2, p-ATR, and p-ATM levels in formalin-fixed paraffin-embedded xenograft specimens derived from *H19*-silenced, ILF2-silenced, and shCtrl MCF-7 cells treated with Olaparib. The values represent the average percentage of DAB-positive cells. The data represent the mean ± s.d. ns, not significant, and *** *p* < 0.001 by two-tailed Student’s *t*-test; *n* = 3 independent experiments. (**D**) IHC detection of p-ATR and p-ATM in formalin-fixed paraffin-embedded xenograft specimens derived from *H19*-silenced, ILF2-silenced, and shCtrl MCF-7 cells treated with Olaparib. Scale bar, 50 μm (**E**) Ki-67 staining (**left**) and TUNEL labeling (**right**) were performed on formalin-fixed paraffin-embedded xenograft specimens derived from *H19*-silenced, ILF2-silenced, and shCtrl MCF-7 cells treated with Olaparib. Scale bar, 100 μm (**F**) Quantification of Ki-67 and TUNEL levels in formalin-fixed paraffin-embedded xenograft specimens derived from *H19*-silenced, ILF2-silenced, and shCtrl MCF-7 cells treated with Olaparib. The data represent the mean ± s.d. ns, not significant, and *** *p* < 0.001 by two-tailed Student’s *t*-test; *n* = 3 independent experiments. (**G**) Final sizes of individual *H19* forced expressed SUM149 cell derived and control xenografts treated with a regimen of 50 mg/kg Olaparib daily for 28 days or left untreated. ns, not significant, and *** *p* < 0.001 by two-tailed Student’s *t*-test. (**H**) Relative xenograft growth of individual SUM149 cells with forced expression of *H19* and control cells treated with a regimen of 50mg/kg Olaparib daily for 28 days or left untreated. Data shown are the mean ± SD. ns, not significant, and *** *p* < 0.001 by ANOVA test; *n* = 4 mice for each group. (**I**) Expression of *H19* was examined by in situ hybridization, and levels of p-ATM and p-ATR were detected by IHC in formalin-fixed paraffin-embedded xenograft specimens. Scale bar, 100 μm (**J**) Quantification of p-ATR and p-ATM levels in formalin-fixed paraffin-embedded xenograft specimens. The values represent the average percentage of DAB-positive cells. The data represent the mean ± s.d. ns, not significant, ** *p* < 0.01, and *** *p* < 0.001 by two-tailed Student’s *t*-test; *n* = 3 independent experiments.

The function of *H19* and ILF2 in regulating xenograft growth and sensitivity to PARP inhibitors was next examined using a xenograft model. *H19* or ILF2-depleted MCF-7 cells and control cells were injected into mammary fat pads of nude mice with pre-implantation of estrogen pellets s.c. followed by Olaparib treatment when the xenografts were palpable. *H19* or ILF2 depletion led to a drastic reduction in tumor volume, suggesting that *H19* and ILF2 promote tumor progression (Figure 7A). Furthermore, *H19* or ILF2 depletion significantly sensitized xenografts to Olaparib treatment (Figure 7B). IHC analysis showed that the xenografts derived from *H19*- or ILF2-depleted cells exhibited increased phosphorylation levels of ATM and ATR (Figure 7C,D) compared to controls. Furthermore, the level of Ki-67 decreased, while the level of TUNEL increased in *H19*- or ILF2-depleted MCF-7 xenografts compared to controls (Figure 7E,F). These data suggest that *H19* or ILF2 depletion potentiates the efficacy of Olaparib in treating ER+ breast cancer xenografts.

Furthermore, the correlation between ILF2 expression and DDR markers in the TCGA database were examined. ILF2 expression was positively correlated with a number of DDR markers, including BLM, CHEK1, and XRCC2 (Figure S3A,B). Moreover, analysis of the UCSC Xena database showed that ILF2 levels were enhanced in the primary cancer compared with paired adjacent normal tissues (Figure S3C,D). Analysis of the Biogrid database revealed that ILF2-interacting proteins are mostly enriched in the DDR signaling pathway, which further supports that the involvement of *H19* and ILF2 in the DNA damage response of cancer cells (Figure S2A–D).

As *H19* or ILF2 depletion was shown above to increase the sensitivity of BRCA1 wild-type MCF-7 cells to PARP inhibitors, it was further determined whether forced expression of *H19* would increase the resistance of BRCA1-mutant SUM149 cells to PARP inhibition. SUM149 cells with forced expression of *H19* or control cells were subsequently injected into mammary fat pads of nude mice, followed by Olaparib treatment when the xenografts became palpable. Forced expression of *H19* did not alter xenograft volume, but significantly increased the resistance of xenografts to Olaparib treatment (Figure 7G,H). Consistently, Olaparib treatment increased, whereas forced expression of *H19* decreased p-ATM and p-ATR levels (Figure 7I,J). These data indicate that forced expression of *H19* confers resistance to PARP inhibition in BRCA1 mutant breast cancer cells.

## 3. Discussion

The treatment of advanced breast cancer generally involves surgery with adjuvant chemotherapy, which improves the progression-free survival of advanced and recurrent BC patients; however, the overall survival of these patients remains invariably poor [40]. Long noncoding RNAs (lncRNA) are a recently identified class of oncogenic drivers, although the functions of lncRNAs in the response to chemotherapeutics or targeted therapies remains largely unclear. This study has provided multiple lines of evidences that the lncRNA *H19* promotes cancer progression and modulates the DNA damage response in ER+ and triple-negative breast cancer cells. It was demonstrated that increased expression of *H19* increases DNA DSB repair, which results in reduced sensitivity to PARP inhibition in breast cancer cells. *H19* was shown to interact directly with ILF2, and it was further demonstrated that *H19* promotes DNA damage repair via ILF2. ILF2 is an RNA-binding protein which has been reported to regulate the splicing of DDR-related genes, thereby affecting the damage response and leading to resistance to chemotherapeutic agents [35]. Consistently, *H19* and ILF2 were further revealed to affect the expression of a series of DNA damage repair-related genes. Although the possible involvement of *H19* in cancer chemotherapy responses has previously been suggested [41,42], definite evidence along with the mechanism by which *H19* promotes DNA damage repair in breast cancer has been provided herein.

TNBC or ER+ breast cancer patients who are BRCA1-deficient are sensitive to DNA-damaging drugs [8]. PARP inhibitors, which are somewhat efficacious in BRCA-deficient breast cancer, enhance the efficacy of chemotherapeutic drugs. However, the incidence of BRCA1/2 mutant germline mutation in TNBC is less than 15% [8]. Thus, it is imperative to employ other mechanisms to promote the downregulation of BRCA1 protein in TNBC. BRCA1 participates in DNA damage repair and influences the ubiquitination of other proteins [43]. Due to the complex diversity of BRCA1 functions, its expression levels are tightly regulated. It is interesting to observe that *H19* and ILF2 promote BRCA1 expression by increasing its stability via the ubiquitin-proteasome proteolytic pathway, possibly via the BRCA1 ubiquitin ligases [38,39] HUWE1 and UBE2T, as suggested in this study. Thus, reduced expression of *H19* or ILF2 might serve as a biomarker for determining breast cancer patients with BRCA1 deficiency or low BRCA1 for consideration for use of PARP inhibitors for adjuvant therapy.

Furthermore, evidence has been presented that *H19* depletion increases sensitivity to PARP inhibitors via ILF2 in ER+ breast cancer cells with wild-type BRCA1 and in BRCA1 mutant triple-negative breast cancer cells. These findings suggest that *H19* inhibition may sensitize breast cancer cells with intact HR repair capacity to PARP inhibitors. *H19* depletion may be used for abrogating the re-establishment of HR caused by 53BP1 deletion, and possibly Shieldin factor deletion or BRCA1/2 reverse mutation [44]. Therefore, further efforts to determine strategies to target *H19* in breast cancer cells are warranted.

In conclusion, this study demonstrated that lncRNA *H19* directly binds to ILF2 and thereby co-regulates DNA damage response and sensitivity to PARP inhibitors in breast cancer. This finding may potentially broaden the scope of application of PARP inhibitors, and suggests that targeting the *H19*/ILF2/BRCA1 axis may potentially serve as a valuable combination therapeutic approach for breast cancer.

## 4. Materials and Methods

### 4.1. Cell Lines

If not specified otherwise, all cell lines used in this study were obtained from ATCC. MCF-7, T47D, HCC1937, and SUM149 cells were authenticated by STR profile analysis. Mycoplasma contamination was tested using RT-PCR routinely. HEK293T was from Dr. Yide Mei (USTC, Hefei, China). MCF-7, T47D, and HCC1937 cells were cultured in RPMI 1640 (Gibco, Waltham, MA, USA) supplemented with 10% fetal bovine serum (Gibco) at 37 °C and 5% CO_2_. SUM149 cells were cultured in Ham’s F12 (Gibco) supplemented with 5% fetal bovine serum (Gibco), 5 μg/mL insulin (Sigma-Aldrich, St. Louis, MO, USA), and 1 μg/mL hydrocortisone (Sigma-Aldrich). HEK293T cells were cultured in DMEM (Gibco) supplemented with 10% fetal bovine serum (Gibco) at 37 °C and 5% CO_2_.

### 4.2. Antibodies and Reagents

Antibody information for this study is provided in Supplementary Table S3. By incorporating short hairpin RNA (shRNA) knockdown experiments into this investigation, the specificity of antibodies against ILF2 and BRCA1 was confirmed. Reagents used in this study: Olaparib, AZD2461 and Doxorubicin (Topscience, Shanghai, China); MG132 and cycloheximide (Selleck, Houston, TX, USA); polyethyleneimine (Polysciences, Warrington, PA, USA); DAPI and Hoechst 33,342 (Sigma-Aldrich).

### 4.3. In Vitro Transcription

The DNA template used for the in vitro synthesis of biotinylated *H19* was amplified by PCR. The forward primer contains the promoter sequence for T7 RNA polymerase, enabling further in vitro transcription. PCR products were purified with the DNA Gel Extraction Kit (AxyPrep, Corning, NY, USA) according to the manufacturer’s instructions and in vitro transcription was conducted with the T7-Flash Biotin RNA Transcription Kit (Epicentre, Madison, WI, USA).

### 4.4. Biotin RNA Pulldown and Mass Spectrometry Assay

Biotin-labeled *H19* was obtained using the T7-Flash Biotin RNA Transcription Kit incubated with breast cancer cell lysate overnight. The samples were sent to ProtTech (Suzhou, China), and *H19*-specific bands were identified using mass spectrometry and aligned to the human proteomic library.

### 4.5. Cytosolic/Nuclear Fractionation

Cytosolic/nuclear fractionation was performed according to the manufacturer’s instructions (Beyotime, Shanghai, China). Briefly, 2 × 10^6^ cells were resuspended in 0.2 mL cytoplasmic extraction reagent A for 10 min incubation, followed by 10 μL cytoplasmic extraction reagent B for 1 min incubation. After centrifugation at 12,000× *g* for 5 min, the supernatant was collected as the cytosolic fraction. Next, the pellets were resuspended in 50 μL nuclear extraction reagent for 30 min incubation. Finally, the nuclear fractionation was collected after centrifugation at 12,000× *g* for 10 min by removal of insoluble debris.

### 4.6. Immunofluorescence Staining

Cells were seeded on coverslips, fixed with 4% paraformalde, permeabilized with 0.1% Triton X-100, and incubated overnight at 4 °C with the following primary antibodies: H2A.X (Cell Signaling, Boston, MA, USA), 53BP1 (Novus, Littleton, CO, USA), ILF2 (Abcam, Cambridgeshire, ENG, UK), and BRCA1 (Immunoway, Plano, TX, USA). Afterwards, secondary antibodies coupled with Alexa 488 or 594 dye were applied, and DAPI or Hoechst 33342 was used to stain nuclei. The quantification of foci was performed as stated previously [45].

### 4.7. Comet Assays

The assays were performed following the directions provided by the manufacturer (Trevigen, Gaithersburg, MD, USA). On the comet slides, low-melting-point agarose was combined with 500 cells (1 × 10^5^ cells per mL) at 37 °C. The slides were placed in a newly made alkaline unwinding solution to allow DNA to unfold for 1 h at 4 °C after being solidified for 10 min at 4 °C. Electrophoresis was then performed on the slides (21 V for 30 min). The slides were subjected to two ddH_2_O washes, five minutes of immersion in 75 percent ethanol, propidium iodide staining, and fluorescence microscopy examination. Utilizing the Comet Assay IV program (Perceptive Instruments, Philadelphia, PA, USA), the comet’s tail DNA content was calculated as a percentage.

### 4.8. Cell Viability

The MTT assay and CCK8 assay were used to determine cell viability and proliferation. Cells were plated in 96-well plates in RPMI 1640 or Hams F12 complete media at a density of 2–4 × 10^3^ per well for MTT tests. On the second day, 10 μL of MTT (5 mg/mL) was added to each well for 2–4 h of incubation at 37 °C before removing the culture media. The purple residue was then dissolved in 100 μL of DMSO and the absorbance at 570 nm was measured. Next, cells were seeded onto 96-well plates for the CCK8 tests (2–4 × 10^3^ cells per well) and allowed to adhere for 12 h. The cells were then given the appropriate doses of Olaparib, AZD2461, or Doxorubicin at 37 °C in the medium. Following treatment, cells were incubated for an additional 2–4 h with the CCK8 reagent at a concentration of 10% in a complete medium. The microplate reader was then used to measure the absorbance at 450 nm. By comparing the absorbance to that of untreated control, it was possible to determine the survival of genotoxin-exposed cells. Three to four replicates were used to produce all of the experimental data. With the aid of GraphPad Prism 8 (GraphPad Prism Software, San Diego, CA, USA), the results were presented graphically.

### 4.9. Immunohistochemistry on Paraffin Sections

For IHC, sections were rehydrated before being incubated for 10 min with 0.3% H_2_O_2_ in water to block endogenous peroxidase. Sections were first treated for 60 min at room temperature with blocking buffer (10% normal goat serum diluted in PBS) and then overnight at 4 °C with a primary antibody diluted in blocking buffer. p-ATM and p-ATR were the primary monoclonal antibodies employed. The sections were incubated with the biotinylated secondary antibody the next day for 60 min at room temperature. Afterward, the sections received the addition of streptavidin/biotin HRP-conjugate for 30 min. The signal was produced utilizing the Vector DAB substrate kit and following the manufacturer’s instructions.

### 4.10. Xenograft Mouse Model

The Institutional Animal Care and Use Committee of the University of Science and Technology of China gave its approval to the design and methodology of animal research. Briefly, 1 × 10^6^ MCF-7 or SUM149 cells were injected orthotopically into the mammary fat pads of 5-week-old female BALB/c nude mice (Slaccas Co., Shanghai, China) using a 1:1 mixture of Matrigel (BD Biosciences, Franklin Lakes, NJ, USA) and MCF-7 or SUM149 cells. A slow-release pellet containing 0.18 mg of 17β-estradiol was subcutaneously inserted into the rear surface of nude mice in orthotopic models for MCF-7 cell-derived xenografts (Innovative Research of America, Sarasota, FL, USA). The mice were divided into test groups randomly when the xenograft reached a maximum diameter of roughly 5 mm. Olaparib (5 mg/kg) or vehicle as control was given i.p. every day. Every three days, the xenograft volume was computed as (length × width^2^)/2. The primary xenografts from the mice were removed and sent for histologic examination five weeks following orthotopic injection.

### 4.11. Lentivirus-Based Transduction of Cells with shRNA

Glycerol stocks of shRNA hairpins were obtained from the Sigma-Aldrich Mission library (TRC 1.0) and the plasmids were isolated by a high pure plasmid Mini Kit (Axygen). HEK293T cells were seeded in 10 cm dishes for 16 h and co-transfected with 12 μg expression vector, 6 μg pSPAX2, and 6 μg pMD2G plasmid using polyethyleneimine (PEI) (Polysciences, Warrington, PA, USA). The medium was refreshed after another 16 h. The supernatant of 293T cells containing lentivirus was collected after 24 h to infect cells with 8 μg/mL polybrene (Sigma-Aldrich) for 24 h. After infection, the supernatant was removed and replaced with a new puromycin medium.

### 4.12. PARP Inhibitor Treatment Study

On day 0, 3 × 10^4^ (SUM149PT or T47D) cells were seeded per well with PARP inhibitor (or untreated control) into six-well plates. Every 3 days, PARP inhibitor was added to the medium of the PARP inhibitor treatment groups. The untreated control group ended on day 6. After a further one to two weeks, the PARP inhibitor therapy groups were discontinued and stained with 0.1% crystal violet on day 6. Using the MCF-7 or HCC1937 cells, 2 × 10^4^ cells were seeded per well with olaparib (or untreated control) into 6-well plates. Every 3 days, Olaparib was replenished in the medium for PARP inhibitor treatment groups. The Olaparib treatment groups were terminated on day 12 and stained with 0.1 percent crystal violet. The untreated control group was halted on day 8. The absorbance of crystal violet at 590 nm was measured to quantify the clonogenic assay.

### 4.13. qRT-PCR

Total RNA was extracted from cultured cell lines using Trizol reagent following the manufacturer’s guideline (Invitrogen, Carlsbad, CA, USA). cDNA synthesis was performed using the TransScript All-in-One First-Strand cDNA Synthesis SuperMix for qPCR with gDNA remover (TransGen Biotech, Beijing, China). The expression levels of analyzed genes were determined using PerfectStart Green qPCR SuperMix (TransGen Biotech).

GAPDH or 18S served as an internal control for normalizing the relative expression of genes in cancer cells. Primers are provided in Supplementary Tables S1 and S2.

### 4.14. Western Blotting

Monolayer cells were lysed with Cell lysis buffer for Western and IP (Beyotime) after being rinsed three times with ice-cold PBS. BCA protein assay (Thermo Fisher Scientific, Waltham, MA, USA) was used to determine protein concentration. Proteins were subjected to 8–12% SDS-PAGE gels for separation and transferred to a nitrocellulose filter membrane (Merck Millipore, Darmstadt, Germany). Membranes were incubated with the primary antibody overnight at 4 °C after blocking for 2 h with 5% non-fat milk and then with the corresponding secondary antibody for 1 h at room temperature. Antibodies used are listed in Supplementary Table S3. After incubation, the membranes were placed in ECL reagent (Thermo Fisher Scientific). Images have been cropped for presentation. Full-size images are shown in the Supplementary Materials.

### 4.15. FISH Analysis

*H19* fluorescence in situ hybridization was performed using a FISH kit (RiboBio, Guangzhou, China) according to the manufacturer’s instructions. Briefly, MCF-7 or SUM149 cells were fixed for 20 min in 4% paraformaldehyde, digested for 30 min, prehybridized for 60 min at 37 °C with hybridization solution, and incubated for overnight at 37 °C with a manufactured cy3-labeled *H19* or Actin FISH Probe Mix. Then, the samples were counterstained with DAPI and observed using confocal microscopy.

### 4.16. RIP Assay

The Magna RIP RNA-binding protein immunoprecipitation kit was used to conduct RIP tests (Merck Millipore). Essentially, 15 cm plates of developing cells were lysed in 1 mL lysis buffer with protease inhibitors and RNase Inhibitor (Thermo Fisher Scientific) and then centrifuged for 20 min at 12,000 rpm. The supernatants were treated with Protein A/G UltraLink Resin (Thermo Fisher Scientific) for 12 h at 4 °C with moderate rotation and the matching antibodies. The beads were washed three times with RNase inhibitor-containing wash buffer. Each antibody’s isotype control (IgG) precipitation and total RNA (input control) were assessed simultaneously. The co-precipitated RNAs were identified using quantitative real-time PCR.

### 4.17. HR Reporter Assay

To establish MCF7-DR-GFP (MCF7-HR) cells, the pDR-GFP plasmid was stably integrated into MCF7 cell lines as previously described. MCF7-DR-GFP cells were transfected using Lipofectamine 3000 (Invitrogen) with 2 μg pCMV-I-SceI. After 48 h of transfection, cells were assessed for green fluorescence emission using flow cytometry. The results were corrected according to the transfection efficiency of the cell line.

### 4.18. Statistical Analysis

Quantitative data were are presented as mean ± SD or SEM. GraphPad Prism 8 or Excel 2013 was applied for statistical analysis. Student’s *t*-test (unpaired and two-tailed) was used to compare two groups, while one-way ANOVA was used to compare data from multiple groups. The Mann–Whitney U-test was used for statistical evaluation of comet assay and colony formation data. Data from Breast Invasive Carcinoma (TCGA, PanCancer Atlas) in the TCGA database were extracted to analyze the Pearson correlations between ILF2 and DDR markers. Data from Breast Invasive Carcinoma (TCGA, PanCancer Atlas) in the TCGA database were extracted to analyze the Pearson correlations between *H19* and several E3 ubiquitin ligases. Except for the experiments presented in Figure 1C–E,G and Figure S3C,D, which were repeated twice, each experiment was performed independently at least three times with similar findings (*p* < 0.05).

## Data Availability

Not applicable.

## Supplementary Materials

The following supporting information can be downloaded at: https://www.mdpi.com/article/10.3390/ijms24119157/s1.
