# Supplementary material for: LncRNA H19 Regulates Breast Cancer DNA Damage Response and Sensitivity to PARP Inhibitors via Binding to ILF2
Source: Int J Mol Sci. 2023 May 23;24(11):9157. doi: 10.3390/ijms24119157 (PMC10252380; doi:10.3390/ijms24119157)
Supplement: Supplementary file 1 [file ijms-24-09157-s001.zip › ijms-2377748-Supplementary Materials.pdf]

Supplementary Materials

Supplementary Figure S1. H19 regulates DNA damage response and sensitivity to PARP inhibitors via binding to ILF2. (A) H19 levels in H19-depleted MCF-7 cells were determined by qRT-PCR. Data are shown as means ± SD. *** $p < 0.001$ by two-tailed Student's *t*-test. Data are representative of at least three independent experiments. (B) The H19 levels in H19-overexpression MCF-7 cells were determined by qRT-PCR. Data are shown as means ± SD. *** $p < 0.001$ by two-tailed Student's *t*-test. Data are representative of at least three independent experiments. (C) Doxorubicin or Olaparib-induced DNA damage in control and T47D cells with forced expression of H19, as measured by Immunofluorescence staining. Representative pictures of 53BP1-positive foci in control and cells with forced expression of H19. Scale bars, 10 μm. One hundred cells were analyzed for each time point. (D) Quantification of the mean grey value of 53BP1-positive foci in control cells and T47D cells with forced expression of H19. Error bars, s.d. ns, not significant, *$p < 0.05$ by two-tailed Student's t-test; $n = 3$ independent cell cultures. (E) Doxorubicin or Olaparib-induced DNA damage in control and T47D cells with forced expression of ILF2, as measured by Immunofluorescence staining. Representative pictures of 53BP1-positive foci in control cells and cells with forced expression of ILF2. Scale bars, 10 μm. One hundred cells were analyzed for each time point. (F) Quantification of the mean grey value of 53BP1-positive foci in control cells and T47D cells with forced expression of ILF2. Error bars, s.d. ns, not significant, * $p < 0.05$, and ** $p < 0.01$ by two-tailed Student's *t*-test; $n = 3$ independent cell cultures. (G) H19-depleted or ILF2-supplemented MCF-7 cells were treated with olaparib followed by CCK8 assay. Error bars, s.d. ** $p < 0.01$ by two-tailed Student's *t*-test; $n = 3$ independent cell cultures. (H) H19-depleted or ILF2-supplemented MCF-7 cells were treated with AZD2461 followed by CCK8 assay. Error bars, s.d. ** $p < 0.01$ by two-tailed Student's *t*-test; $n = 3$ independent cell cultures.

Supplementary Figure S2. H19 and ILF2 affect the expression of genes involved in DNA damage response and drug resistance. (A and B) Significantly enriched pathways by multiple interacting proteins of ILF2 in the BioGRID database. (C and D) Significantly enriched pathways by multiple

interacting proteins of ILF2 in the BioGRID database. (E) GSEA plots of ABC transporters-related signatures in H19-depleted or ILF2-depleted MCF-7 cells versus control cells. Abbreviations: FDR, false discovery rate; NES, normalized enrichment score. (F) GSEA plots of doxorubicin resistance-related signatures in H19-depleted or ILF2-depleted MCF-7 cells versus control cells. Abbreviations: FDR, false discovery rate; NES, normalized enrichment score. (G and H) GSEA plots of p53 pathway-related and UV response-related signatures in H19-depleted or ILF2-depleted MCF-7 cells versus control cells. Abbreviations: FDR, false discovery rate; NES, normalized enrichment score.

Supplementary Figure S3. ILF2 is associated with DNA damage in breast cancer. (A) Pearson correlation between ILF2 expression and expression of BLM, CDC25C, CHEK1, CHEK2, EXO1, FANCA, FANCD2, FANCG and FEN1 in the breast cancer samples (1082 samples in which ILF2 expression was detectable were used in correlation analysis). (B) Pearson correlation between ILF2 expression and expression of MSH2, PARP1, PCNA, RAD51, RNF8, RPA1, UNG and XRCC2 in the breast cancer samples (1082 samples in which ILF2 expression was detectable were used in correlation analysis). (C) ILF2 expression was enhanced in breast cancer samples from the TCGA cohort (n = 112 biologically independent samples) compared with paired adjacent normal tissues; $p < 0.001$ by two-tailed Student's t-test. (D) ILF2 expression was enhanced in breast cancer samples from the TCGA cohort (n = 114 biologically independent samples) compared with paired adjacent normal tissues; $p < 0.001$ by two-tailed Student's t-test.

Supplementary Table S1. Primers and probes used in this study.

Supplementary Table S2. Primers and probes used in this study.

Supplementary Table S3. Antibodies used in this study.
